# The Role of MiR-5094 as a Proliferation Suppressor during Cellular Radiation Response via Downregulating STAT5b

**DOI:** 10.7150/jca.39679

**Published:** 2020-02-03

**Authors:** Nan Ding, Junrui Hua, Jinpeng He, Dong Lu, Wenjun Wei, Yanan Zhang, Heng Zhou, Liying Zhang, Yongqi Liu, Guangming Zhou, Jufang Wang

**Affiliations:** 1 Key Laboratory of Space Radiobiology of Gansu Province & Key Laboratory of Heavy Ion Radiation Biology and Medicine of Chinese Academy of Sciences, Institute of Modern Physics, Chinese Academy of Sciences, Lanzhou 730000, China; 2 Gansu University of Traditional Chinese Medicine, Lanzhou 730000, China; 3 Medical College of Soochow University, Suzhou 215123, China

**Keywords:** microRNA, radiation, miR-5094, STAT5b, proliferation

## Abstract

MicroRNAs (miRNAs) play important roles in the regulation of cellular stress responses. We previously uncovered 10 novel human miRNAs which are induced by X-ray irradiation in HeLa cells using Solexa deep sequencing. The most highly expressed new miRNA, miR-5094, was predicted to target STAT5b. This study wonders whether miR-5094 participates in cellular radiation response via STAT5b. Firstly, direct interaction between miRNA-5094 and the STAT5b 3'-UTR was confirmed by luciferase reporter assay. Then, the radiation responsive expression of miR-5094 and STAT5b were measured in HeLa and Jurkat cells, and the expressions of down-stream genes of STAT5b after ionizing radiation (IR) were detected in HeLa cells. At last, the effects of miR-5094 on survival fraction, cell proliferation, cell cycle arrest and apoptosis induced by IR were investigated in HeLa cells, Jurkat cells and human peripheral blood T cells. It was found that up-regulation of miR-5094 by radiation induction or miRNA mimic transfection suppressed expression of STAT5b, and consequently decreased the transcription of down-stream Igf-1 and Bcl-2. Additionally, over expression of miR-5094 resulted in proliferation suppression and knockdown of miR-5094 by miRNA inhibitor after irradiation partially reversed the proliferation suppression induced by miR-5094 in HeLa cells, Jurkat cells and CD4^+^ T cells. Collectively, our findings demonstrate that up-regulation of miR-5094 down-regulated the expression of STAT5b, thereby suppressing cell proliferation after X-ray irradiation.

## Introduction

Radiation therapy (RT) is one of the three primary modalities of cancer treatment. RT uses ionizing radiations (IR) to cure tumor patients by IR-induced direct and indirect DNA damages, on the basis of classic target theory. Mammalian cells respond to IR-induced DNA damages by activating sophisticated DNA damage response (DDR), which includes cell cycle arrest, activation of DNA repair genes, and triggering of apoptosis 1.

RT also modulates the tumor microenvironment 2. In recent studies, RT has been demonstrated to induce the activation of T cells, which in turn cause immunogenic cell death of cancer cells 3. Growing number of researches described the combination of radiotherapy with immunotherapy. For instance, immune checkpoint inhibitors (ICI), make it possible to enhance antitumor effects 4, 5. It is important to consider the biology of radiation response of immune system in developing strategies of combined radiotherapy with immunotherapy.

Up-regulation and down-regulation of microRNAs (miRNAs) represent unique cellular radiation responses. MiRNAs are a class of endogenous, short (~22 nucleotides) and single-stranded non-coding RNAs, which act as negative regulators of gene expression at post-transcriptional level. MiRNAs bind to the 3' untranslated region (3'UTR) of mRNA of their targets, and subsequently promoting mRNA degradation and/or inhibiting translation 6. As powerful post-transcriptional regulators, miRNAs are broadly involved in regulation of radiation-related damage repair processes 7-9. In last two decades, a larger number of radiation responsive miRNAs were detected by miRNA microarray and deep sequencing technology 10-13. A lot of radiation responsive miRNAs were confirmed to regulate cellular radiation sensitivity by manipulating expression of DNA damage repair regulators 11-13. For example, a p53-dependent up-regulation of miR-34 after irradiation was reported both *in vivo* and *in vitro*, and, the miR-34 down-regulated Bcl-2 and Notch, which in turn sensitized cancer cells to radiation 14, 15. In our previous study, 190 up-regulated miRNAs and 231 down-regulated miRNAs were detected in HeLa cells after 2 Gy X-ray irradiation using Solexa sequencing, and 10 novel human miRNAs were identified by qRT-PCR 16. Among these 10 miRNAs, miR-5094 was the most abundantly expressed one after radiation treatment, which was predicted to target signal transducers and activators of transcription 5b (STAT5b) 16.

STAT5b is an important member of the *Janus* kinase/signal transducers and activators of transcription (JAK/STAT) signaling pathway which plays key biological roles in growth, immune responses and cancers 17, 18. As a universal transcription factor, STAT5b is stimulated by various cytokines including growth hormones (GH) and interleukins 19. Particularly, STAT5b is a key mediator of GH-regulated Igf-I transcription which in turn influence cell growth both *in vivo* and *in vitro* 20. STAT5b was also reported to regulate cell growth, cell cycle progression and apoptosis in human glioblastoma and breast cancer cells through its down-stream genes like Bcl-2, p21 and Cyclin D1 21-23. Furthermore, CD4^+^ and CD8^+^ T cells from the STAT5b transgenic NOD mice showed a higher proliferation capacity, and the number and proportion of CD4^+^CD25^+^ regulatory T cells were significantly increased 24. Conversely, in STAT5b-deficient patient, deficiency of STAT5b function displayed immune dysregulation and decreased numbers of CD4^+^CD25^high^ T cells 25.

Several studies reported that the expression and activation of STAT5b were modulated by IR and UV radiation (UVR). It was demonstrated that UVR inhibited IL-2-induced activation of STAT5 in T lymphocytes 26. Likewise, down-regulation of STAT5b expression was detected in ATM mutated human fibroblast AT5BIVA cells at half hour after 10 Gy γ-ray irradiation 27. However, up-regulation of STAT5b was detected in peripheral blood of neuroblastoma patients 72 h after Medical Internal Radiation Dose (MIRD) schema 28. Also, the expression of STAT5b was slightly increased in both directly irradiated and bystander k562 cells at one hour after 4 Gy of X-ray irradiation 29. Yet, the underlying mechanism of STAT5b aberrant expression during cellular radiation response is unknown.

Several miRNAs have been described to target STAT5b. It was reported that miR-200a directly repressed STAT5b expression in both mice and human 30, while miR-134 was showed to inhibit proliferation, survival and xenograft growth in cancer cell and stem-cell by targeting STAT5b and KRAS 31. Similarly, miR-150 was demonstrated to suppress expression of STAT5b in mammary epithelial cells of bitransgenic mice 32. Whether and how miRNAs regulate STAT5b in DNA damage response to IR remains to be an open question.

In present study, we validated STAT5b as a target of miR-5094. We demonstrated an inhibition of STAT5b expression by radiation-induced miR-5094 and uncovered suppressing effect of miR-5094 on proliferation of different cells, especially on proliferation of CD4^+^ T cells and CD4^+^CD25^+^ Treg cells during cellular radiation response. Our study for first time provided evidences that radiation- induced miR-5094 participates in cellular radiation response via targeting STAT5b.

## Materials and Methods

### Cell lines

Human cervical carcinoma cell line HeLa, Epstein-Barr virus transformed B cells (EBV-B) and human acute T cell leukemia Jurkat cells were cultured in RPMI-1640 medium (Gibco, 31800-105, USA), while human bronchial epithelial cell Beas-2B was cultured in DMEM medium (Gibco, 12800-082). Both RPMI-1640 and DMEM medium were supplemented with 10% fetal bovine serum (Gibco, 10099-141), 100 units/mL penicillin and 100 mg/mL streptomycin, and, the cells were maintained at 37℃ in a humidified atmosphere of 95% air and 5% CO_2_.

### The generation of CD4^+^ T cells and CD4^+^CD25^+^ Treg cells

The study protocol was approved by the medical ethics committee of affiliation hospital of Gansu University of Chinese Medicine and the informed written consents were obtained from all volunteers. Peripheral blood was obtained from healthy volunteers and the peripheral blood mononuclear cells (PBMCs) were isolated by density centrifugation over Ficoll (Histopaque 1077, Sigma Aldrich, USA). CD4^+^ T cells were enriched with an isolation kit (CD4^+^ T Cell Isolation Kit human, Miltenyi Biotec, Auburn, CA, USA), which contains a cocktail of CD8a, CD14, CD15, CD16, CD19, CD36, CD56, CD123, TcRγ/δ, and CD235a (Glycophorin A) antibodies. The cell separation was performed with LS columns (Miltenyi Biotech), according to the manufacturer's instructions. The CD4^+^ cells were further labeled with PE-conjugated anti-CD25 mAb (BD Bioscience, CA 555432, USA), PerCP-conjugated anti-CD4 mAb (BD Bioscience, 566321) and FITC-conjugated anti-CD127 mAb (BD Bioscience, 560549). The CD4^+^CD25^+^ Treg cells were obtained by cell sorting (purity > 95%) on MoFlo XDP (Beckman coulter, Pasadena, CA, USA). CD4^+^ cells and CD4^+^CD25^+^ cells were stimulated with anti-CD3/CD28-coated beads (Gibco, 11161D) (1:4), IL-2 (Gibco, PHC0026) (20 U/mL) in AIM-V (Gibco, 12055083) serum-free medium in 12-well plates (BD Labware).

### Cell transfection and irradiation

For HeLa and Beas-2B cells, 2×10^5^ cells were seeded in 35mm dishes and cultured for 24 h prior to be transfected with small RNAs including miR-5094 mimics (GeneCopoeia, HmiR1458, China), negative control (GeneCopoeia, CmiR0001-MR04), miR-5094 inhibitor (GeneCopoeia, HmiR-AN2605), and the inhibitor negative control (GeneCopoeia, CmiR-AN0001-AM01) or STAT5b siRNA (RioNeer, 1145682, 1145685, 1145687, CA, USA) and its negative control (BioNeer, Daejeon, Korea) at 40-60% confluence using Lipofectamine TM 2000 (Invitrogen, 11668-019, USA). Five hours after transfection, the cells were irradiated with a laboratory RX-650 X-ray source (Faxitron, Tucson, Arizona, USA), at a dose rate of 0.8 Gy/min (100 keV, 5mA). For EBV-B cells, Jurkat cells, CD4^+^ T cells and CD4^+^CD25^+^ Treg cells, 5×10^5^ cells in 0.5 mL OPTI-MEM were transfected with different RNAs using Entranster^TM^-R4000 transfection reagent 33 (EnGreen Biosystem, 4000-4, Beijing, China) in 1.5 mL Eppendorf tube. The transfection was done for 5 h with the final concentration of the miRNA mimics or inhibitor at 30 nM, and the siRNAs concentration were at 50 nM. The transfected cells were irradiated at a dose rate of 0.8 Gy/min (100 keV, 5mA). At last, cells were centrifuged and reseeded in 12-well plates with fresh culture medium.

### Luciferase reporter assay

STAT5b 3'-UTRs containing wild type or mutant miR-5094 binding sites, were synthesized by Sangon Biotechnology Co. (Shanghai, China). The segments were inserted into pmirGLO Dual-Luciferase miRNA Target Expression Vectors (Promega, WI, USA). For luciferase reporter assay, 1.5×10^5^ HeLa cells were seeded and grown in 12-well plate for 24 h, and then the cells were co-transfected with 300 ng DNA (pmirGLO-3′ UTR constructs or derived mutants) and 30 nM of miR-5094 mimics (GeneCopoeia, HmiR1458) or negative control (GeneCopoeia, CmiR0001-MR04) using Lipofectamine TM 2000 (Invitrogen, 11668-019). Dual luciferase assays were performed 24 h after transfection using Dual Luciferase Reporter Assay Kit (Promega, E1910) with Tecan Infinite M200 Pro microplate reader (Tecan, Mannedorf, Switzerland).

### qRT-PCR

Total RNA was extracted using TRIzol reagent (Invitrogen, 15596018). Reverse transcription of miRNA or mRNA was conducted with All-in-OneTM miRNA First-Strand cDNA Synthesis kit (GeneCopoeia, QP014) or All-in-OneTM First-Strand cDNA Synthesis kit (GeneCopoeia, AORT-0060). To quantify the miR-5094 expression or mRNA expression of STAT5b, Bcl-2, Cyclin D1, Igf-1 and p21, real-time PCR was performed on CFX96 Touch™ Real-Time PCR Detection System (Bio-Rad, California, USA) using All-inOneTM miRNA qPCR kit (GeneCopoeia, AMPR-0200) or Allin-OneTM mRNA Detection kit (GeneCopoeia, AOPR-0200) based on SYBR-Green. PCR primers for U6 (HmiRQP9001), miR-5094 (HmiRQP2605), miR-34a (HmiRQP0439), miR-134 (HmiRQP3060), miR-150-5p (HmiRQP0209), miR-200a (HmiRQP0297), GAPDH (HQP006940), STAT5b (HQP017774), Bcl-2 (HQP016211), Cyclin D1 (HQP016204), Igf-1 (HQP009518) and p21 (HQP000331) were all purchased from GeneCopoeia. In each qRT-PCR, snRNA U6 and GAPDH mRNAs were used as control for normalization respectively. The relative expression was calculated using the 2^-ΔΔCt^ method.

### Western blotting analysis

Protein lysates were made in RIPA buffer (Beyotime, P0013C, China). Proteins were separated by 12% SDS-PAGE electrophoresis and transferred to a methanol-activated PVDF membrane (Millipore, IPVH00010, USA). The membrane was blocked with 5% milk in PBS containing 0.1% Tween-20 for 1 h and subsequently probed with anti-STAT5b antibody (Abcam, ab226193, MA, USA), anti-pSTAT5b antibody (Abcam, ab52211), anti-beta Actin antibody (Abcam, ab228001), anti-Bcl2 antibody (Abgent, AP20322c, China), anti-Cyclin D1 antibody (Abcam, ab134175), anti-Igf1 antibody (Abgent, AP14099b) or p21 antibody (Santa Cruz, sc-397) at room temperature for 2 h. After 1 h incubation with goat-anti-rabbit HRP-conjugated secondary antibody (Abcam, ab97051) or goat-anti-mouse HRP-conjugated secondary antibody (Abcam, ab97023), the protein bands were detected with luminal reagent (Millipore, WBKLS0500, USA).

### Colony formation assay

HeLa cells were irradiated with 2 Gy of X-rays right after RNA-transfection. Then, cells were harvested by trypsinization and then re-suspended in RPMI-1640 medium supplemented with 10% FBS. An appropriate number of cells were plated into a 60 mm dish to produce ~50-150 colonies. After grown for 10 days, the cells were fixed with 75% ethanol and stained with 0.5% crystal violet at room temperature. The cell colonies containing more than 50 cells were counted and at least three parallel dishes were scored for each treatment. The experiments were conducted independently for more than three times.

### Cell Viability assay

Cell viability was determined by MTT assay. Briefly, HeLa cells were seeded into 96-well plates a day before RNA transfection. The RNA-transfected cells were exposed to 2 Gy of X-rays and allowed to grow for 48 h. Then, 10 µL of MTT solution at 0.5 mg/ mL was added in wells and incubated for 4 h at 37°C. The medium was replaced with 100 µL dimethylsulfoxide and vortexed for 10 minutes. The optical density (OD) values at 570 nm were determined by Tecan Infinite M200 Pro microplate reader.

### Cell growth curves

2×10^5^ Jurkat cells, CD4^+^ T cells or CD4^+^CD25^+^ Treg cells were exposed to 2 Gy of X-rays after RNA transfection, and then centrifuged and reseeded in 12-well plates with fresh medium. CD4^+^ T cells or CD4^+^CD25^+^ Treg cells were stimulated with anti-CD3/CD28 and IL-2 at same time. The cell growth curves were plotted with the cell numbers counted by Coulter Counter (Beckman, Brea, CA, USA).

### Flow cytometry

For cell cycle assay, cells were first exposed to IR at a proper dose. Twenty-four hours post-irradiation, the cells were then fixed in -20°C prechilled 70% alcohol overnight, and stained with 20 μg/mL propidium iodide (PI) at room temperature. For apoptosis assay, cells were harvested 24 h post-irradiation and stained with Annexin V-FITC/PI (BD Biosciences, 556547) for 30 min at room temperature. For CD4^+^CD25^+^ Treg percentage assay, cells were labeled with PE-conjugated anti-CD25 mAb and FITC-conjugated anti-CD4 mAb (BD Bioscience, 550628).

Flow cytometry assay was performed using an Amnis imaging flow cytometer (Merck Millipore, Darmstadt, Hesse, Germany) and at least 10,000 gated events were acquired from each sample. The data were analyzed with IDEAS Application v6.0 (Amnis) or FlowJo v6.0 (Tree Star, Ashland, OR, USA).

### Statistical analysis

All the experiments were repeated at least three times and results are shown as mean ± SD. The statistical significance of the results was determined by Student's t-test and P < 0.05 was considered as a statistically significant difference.

## Results

### MiR-5094 directly targets STAT5b

**Figure 1:**
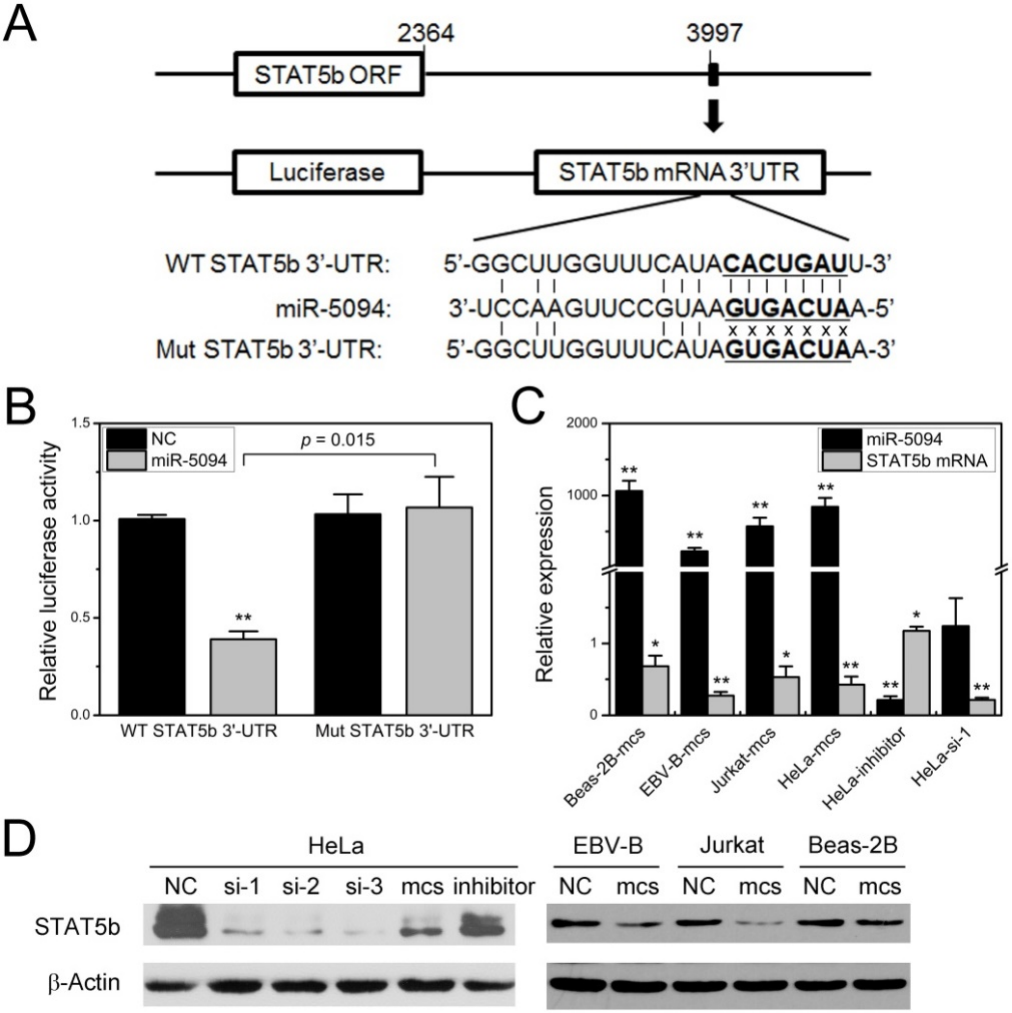
** MiR-5094 directly targets STAT5b. (A)** Alignment of wild-type seed sequence of the 3'-UTR of STAT5b mRNA (WT STAT5b 3'-UTR) and a mutated seed sequence of the miR-5094-binding site (Mut STAT5b 3'-UTR). The seed region is shown in bold. **(B)** Luciferase reporter assays. Luciferase reporter containing wild-type or mutant STAT5b 3'UTR was co-transfected with exogenous miR-5094 mimics (miR-5094) or negative mock control (NC) into HeLa cells. Luciferase activity was measured 24 h after transfection. Renilla luciferase activity was used to normalize the firefly luciferase activity. **(C)** MiR-5094 suppresses STAT5b mRNA expression in different cells at 24 h after transfection. The relative expression levels were normalized to same cells transient transfected with NC at same time point. **(D)** MiR-5094 suppresses STAT5b protein expression in different cells at 24 h after transfection. Mcs: miR-5094 mimics; inhibitor: miR-5094 inhibitor; si-1, si-2 and si-3: STAT5b siRNA. *P < 0.05 and **P < 0.01 represent the comparison with NC.

We previously demonstrated the up-regulation of miR-5094 induced by ionizing radiation and predicted the STAT5b as a targets of miR-5094 by TargetScan software 16. In this study, to determine whether miR-5094 directly targets STAT5b via such a 3′-UTR, we synthesized two kinds of STAT5b 3'-UTR fragments which contain either wild-type or mutated putative miR-5094 seed region (Figure 1A), then cloned them into pmirGLO luciferase report vectors and conducted the reporter gene assay respectively. Co-transfection of luciferase reporter containing wild type STAT5b 3'-UTR and miR-5094 mimics into HeLa cells significantly repressed the luciferase activity by approximately 60% (*P* = 0.015), while suppression of luciferase activity was abolished when a mismatch mutation was introduced in the putative binding sites of STAT5b 3'-UTR (Figure 1B).

Next, we validated the inhibition of STAT5b expression by miR-5094. As shown in Figure 1C and 1D, the miR-5094 mimics specifically suppressed both the STAT5b protein and mRNA expressions at 24 h post-transfection in HeLa cells, Beas-2B cells, EBV-B cells and Jurkat cells. Transient transfection of HeLa cells with miR-5094 inhibitor suppressed expression of miR-5094 and resulted in an increasing of STAT5b mRNA (Figure 1C). As expected, HeLa cells transfected with STAT5b siRNA showed remarkably decrease in STAT5b expression in both transcriptional levels (Figure 1C) and translational levels (Figure 1D).

### Ionizing radiation-induced miR-5094 expression results in STAT5b suppression

**Figure 2:**
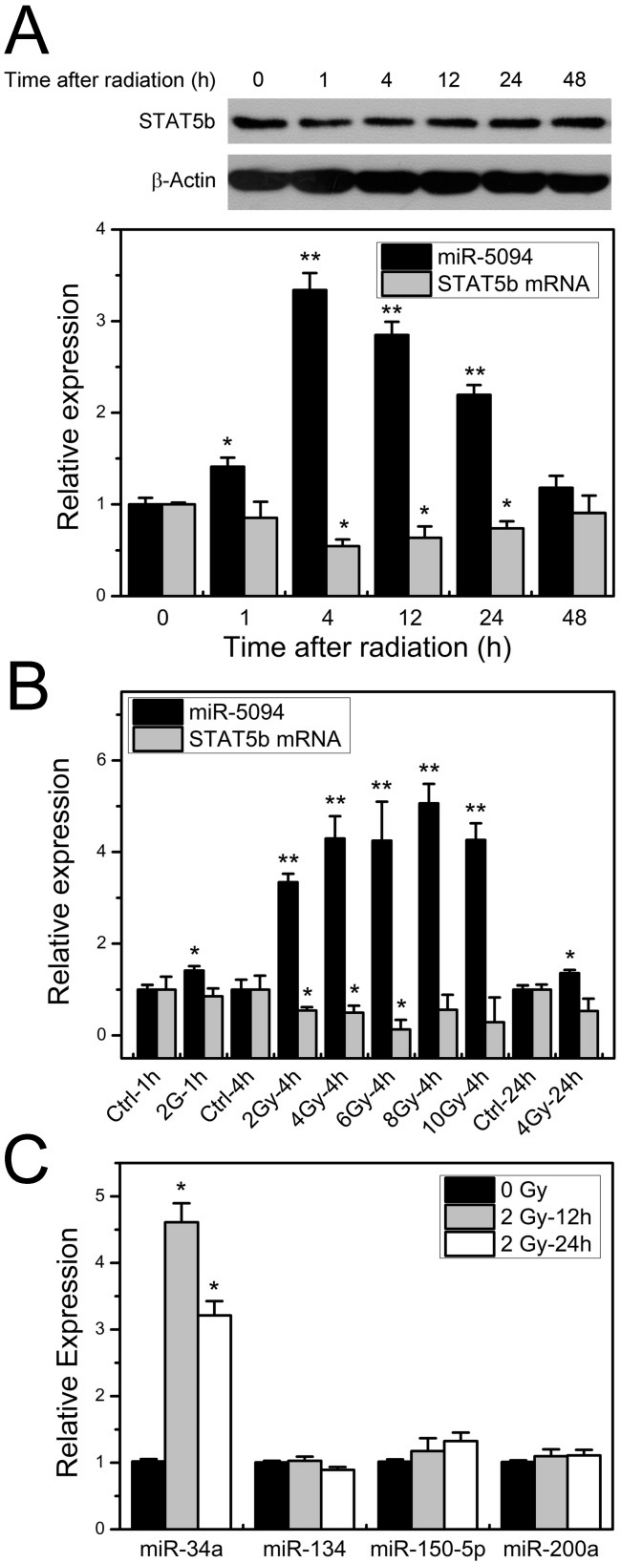
** Radiation induces increase expression of miR-5094 and decrease expression of STAT5b. (A)** STAT5b and miR-5094 expression in HeLa cells at different time points after radiation. U6 was used as control of miR-5094 expression, and GAPDH mRNA was used as control of STAT5b mRNA. **(B)** Expression of miR-5094 and STAT5b mRNA in HeLa cells after different dosages of irradiation treatment. U6 and GAPDH were used as controls. **(C)** Expression of miR-34a, miR-134, miR-150-5p and miR-200a after radiation in HeLa cells. The qRT-PCR was conducted to quantify the expression levels of miR-34a, miR-134, miR-150-5p and miR-200a at 12 h and 24 h after 2 Gy X-rays. U6 was used as controls. *P < 0.05 or **P < 0.01 represents statistical significance of comparison against non-irradiated control.

To investigate the expression profiles of miR-5094 and STAT5b under ionizing irradiation, the kinetics of miR-5094 or STAT5b expression was monitored by quantitative RT-PCR and Western blotting in 2 Gy X-ray irradiated HeLa cells. Expression of miR-5094 increased immediately after radiation and peaked at about 4 h after IR treatment, then declined until 48 h. Levels of STAT5b mRNA and protein decreased gradually after irradiation and the lowest point was detected at about 4 h (Figure 2A). We further examined miR-5094 and STAT5b mRNA expression under different radiation dosages. As shown in Figure 2B, a clear increase in miR-5094 and decrease of STAT5b were detected under all tested doses. At 4 h, the expression of miR-5094 increased with the rising of radiation dose, and peaked at about 8 Gy. Nevertheless, the decrease of STAT5b did not show a clear dose response.

We also checked expression of miR-134, miR-150-5p and miR-200a, which reportedly regulate STAT5b expression 30-32 in X-ray irradiation HeLa cells. The expression of miR-34a, who is up-regulated after irradiation 14, was used as a reference of radiation treatment. As shown in Figures 2C, among miR-134, miR-150-5p and miR-200a, only miR-150-5p was slightly increased but without statistical significance. On the other hand, the expression of miR-34a was significantly increased at both 12 h and 24 h after irradiation.

### MiR-5094 changes the expression of STAT5b down-stream regulators

**Figure 3:**
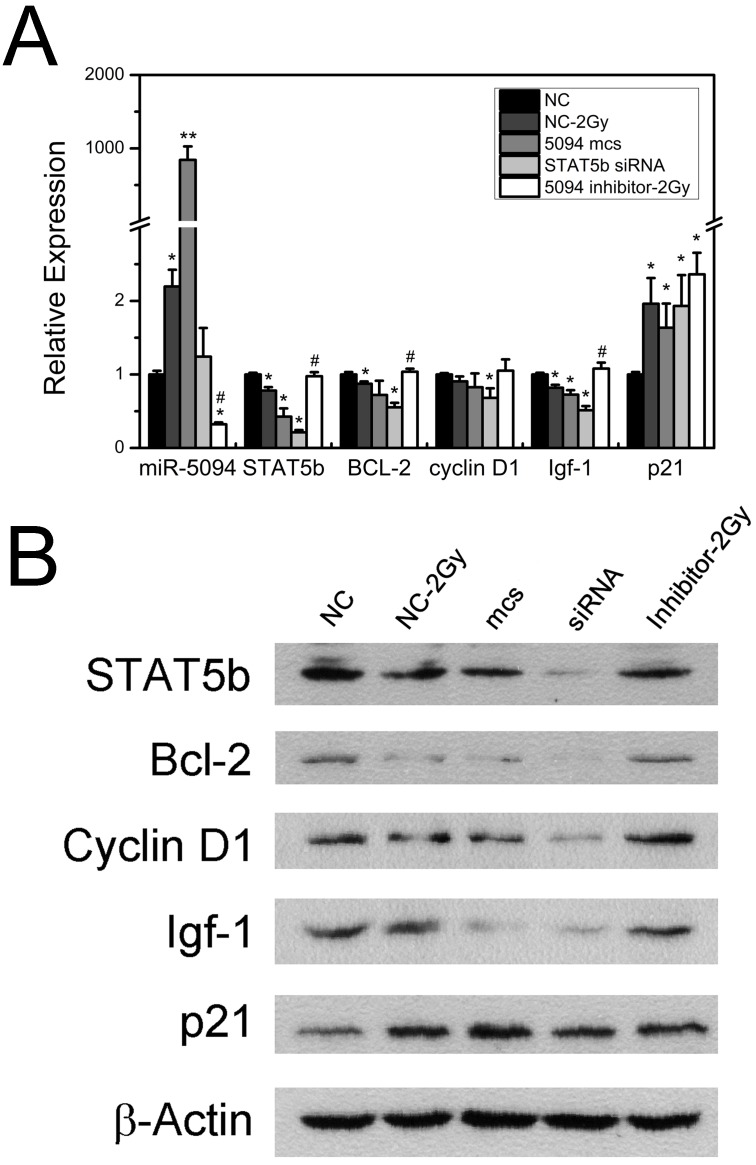
** Influence of miR-5094 on expression of STAT5b and its down-stream genes. (A)** Expression of miR-5094 and mRNA levels of STAT5b, Bcl-2, Cyclin D1, Igf-1 and p21 in HeLa cells at 24 h after radiation. U6 and GADPH were used as controls. **(B)** Protein expression of STAT5b, Bcl-2, Cyclin D1, Igf-1 and p21 in HeLa cells at 24 h after radiation. NC: Negative mock control; 5094 mcs: miR-5094 mimics. *P < 0.05 and **P < 0.01 represent the comparison with NC, while #P < 0.05 represent the comparison with NC plus 2 Gy X-rays (NC-2Gy).

To investigate whether miR-5094 up-regulation affects the expression of STAT5b down-stream genes, we examined expression of Bcl-2, Cyclin D1, Igf-1 and p21, which are known to be regulated by STAT5b and have important roles during cell radiation response 20-22. As shown in Figure 3A, 2 Gy X-rays increased the expression of miR-5094 and decreased the transcription of STAT5b mRNA. Meanwhile, transcription of Bcl-2 and Igf-1 was clearly decreased after transient transfection of miR-5094 mimics or STAT5b siRNA and IR radiation. The Cyclin D1 mRNA was significantly affected by STAT5b siRNA but not by miR-5094 mimics or radiation. Moreover, the expression of p21 was remarkably up-regulated by X-ray irradiation, or by miR-5094 mimics or STAT5b siRNA transfection. More importantly, miR-5094 inhibitor rescued the decline of STAT5b after radiation treatment, and subsequently upregulated the expression of down-stream Bcl-2 and Igf-1, but not significantly influenced expression of Cyclin D1 and p21 (Figure 3A).

Furthermore, translation levels of these genes were also detected. As shown in Figure 3B, radiation treatment suppressed the expression of STAT5b, and down-regulated expression of Bcl-2, Cyclin D1 and Igf-1. On the contrary, the expression of p21 was up-regulated by radiation. As expect, the transfection of miR-5094 mimics or STAT5b siRNA resulted in a down-regulation of STAT5b, Bcl-2, Cyclin D1 and Igf-1, and up-regulation of p21. Moreover, the transfection of miR-5094 inhibitor rescued the expression of STAT5b, Bcl-2, Igf-1 and Cyclin D1 after radiation compared with NC.

### MiR-5094 dampen down proliferation after radiation

To investigate the role of miR-5094 in regulation of the cellular response to IR, the intracellular miR-5094 levels were up/downregulated by transient transfection with miR-5094 mimics (mcs) or inhibitor. Cell survival, cell viability, cell cycle distribution, cell proliferation and apoptosis were monitored after IR.

**Figure 4:**
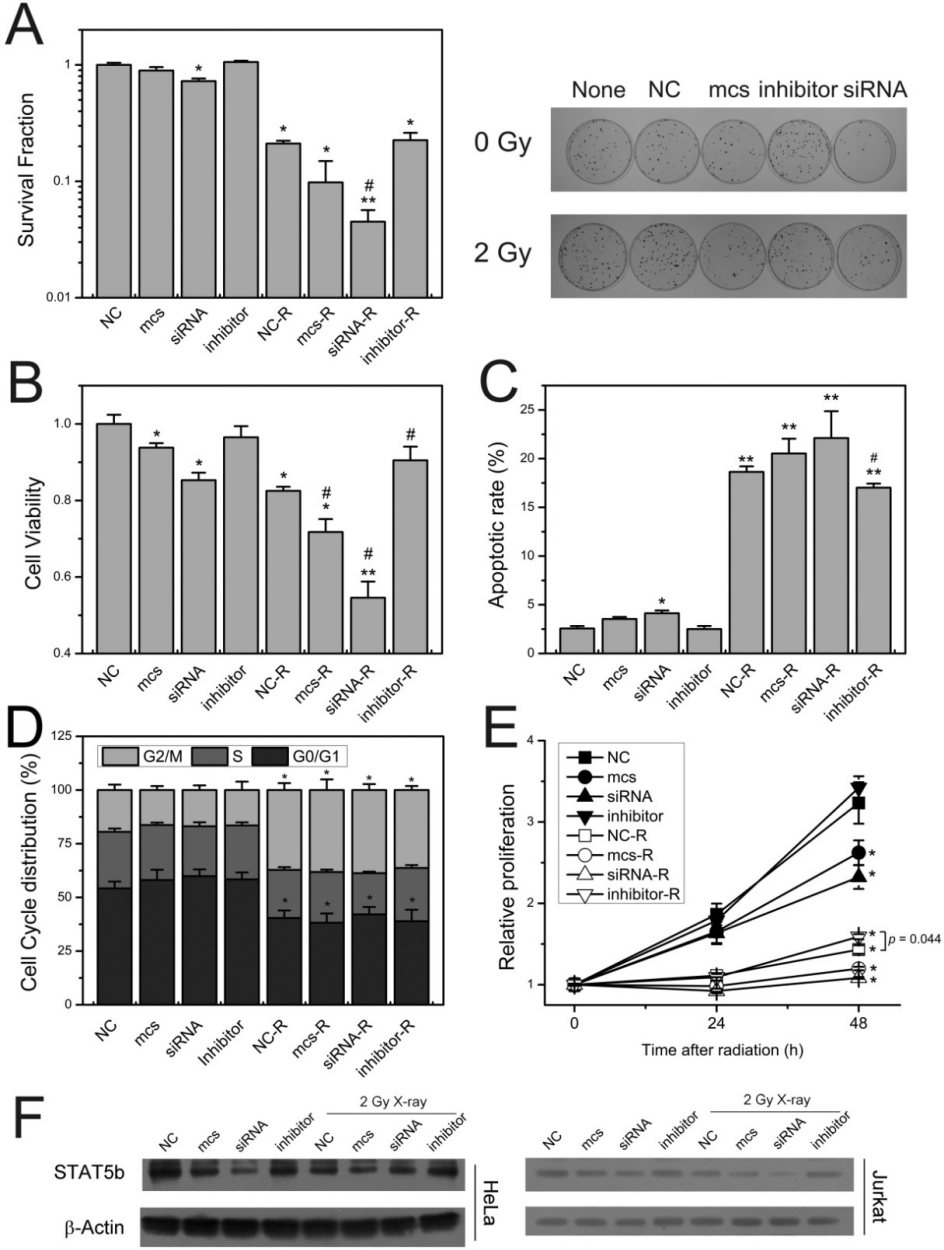
** miR-5094 regulates cellular radiation response by targeting STAT5b. (A)** Survival fraction of HeLa cells. Left graph shows quantitative bar of colony formation assay; right graph is plates of colony formation assay**. (B)** Cell viability assay of HeLa cells at 48 h after 2 Gy X-rays. **(C)** The apoptotic rate of HeLa cells at 24 h after 2 Gy X-rays. **(D)** Cell cycle distribution of HeLa cells treated with 2 Gy of X-rays at 24 h post-irradiation. **(E)** Proliferation curves of Jurkat cells. **(F)** The expression of STAT5b in HeLa cells and Jurkat cells at 24 h after radiation. None: non-transfected control; NC: negative mock control; mcs: miR-5094 mimics; siRNA: STAT5b siRNA; inhibitor: miR-5094 inhibitor. *P < 0.05 and **P < 0.01 represent comparison with NC; #P < 0.05 represent comparison with NC plus 2 Gy X-rays (NC-R).

As shown in Figure 4A, compared with non-irradiated groups, X-ray irradiation significantly decreased HeLa cells survival fraction. Transfection of miR-5094 mimics or STAT5b siRNA remarkably exacerbated the decrease of cell survival after X-ray irradiation, while miRNA inhibitor showed no significant effect on cell survival. Meanwhile, the transfection of STAT5b siRNA induced a moderate decrease of cell survival even without X-ray irradiation.

The cell viability assay showed that radiation markedly suppressed cell viability of HeLa cells. Moreover, like STAT5b siRNA, miR-5094 mimics significantly decreased cell viability, and exaggerated the decrease of cell viability after radiation. Notably, the inhibition of miR-5094 by miRNA inhibitor significantly recovered the decrease of cell viability, as compared with NC transfection group after irradiation (Figure 4B).

The apoptotic assay showed that X-ray irradiation induced significant increase in apoptosis of HeLa cells. Transfection of STAT5b siRNA resulted in an increasing of apoptosis in un-irradiated HeLa cells and exacerbated the apoptosis of HeLa cells after X-ray irradiation (Figure 4C). As compared with negative control, miR-5094 mimics also significantly increased apoptosis of HeLa cells after X-ray irradiation, while less effective than STAT5b siRNA. The miR-5094 inhibitor transfection did not affect non-irradiated cells. However, the inhibitor decreased apoptosis rate of cells compared with negative control after radiation (Figure 4C).

The cell cycle assay showed that X-ray irradiation induced significant G2/M arrested (Figure 4D). However, the transfection of miR-5094 mimics, STAT5b siRNA or miR-5094 inhibitor showed no significant influence on cell cycle distributions with or without radiation treatment (figure 4D).

We also checked the growth of Jurkat T cells. As shown in Figure 4E, X-ray irradiation significantly suppressed cell proliferation of Jurkat cells. Transfection of miR-5094 mimics or STAT5b siRNA remarkably decreased Jurkat cells proliferation rate. However, miRNA inhibitor significantly improved cell proliferation, as compared with negative mock control, especially at 48 h post-irradiation.

We further validated STAT5b protein level in Jurkat T cells and HeLa cells. As shown in Figure 4F, in both HeLa and Jurkat T cell lines, miR-5094 mimics or STAT5b siRNA lowered expression of STAT5b with or without irradiation. Also, the inhibitor of miR-5094 resulted in upregulation of STAT5b.

### MiR-5094 suppresses human peripheral blood T cell proliferation

Because of the key roles of STAT5b and Igf-1 on lymphocyte proliferation and the maintenance of immune function, we further investigate the effect of miR-5094 in human peripheral blood T cells. CD4^+^ T cells and CD4^+^CD25^+^ Treg cells were separated from human peripheral blood and their growth curves were measured after irradiation and miRNAs transfection.

**Figure 5:**
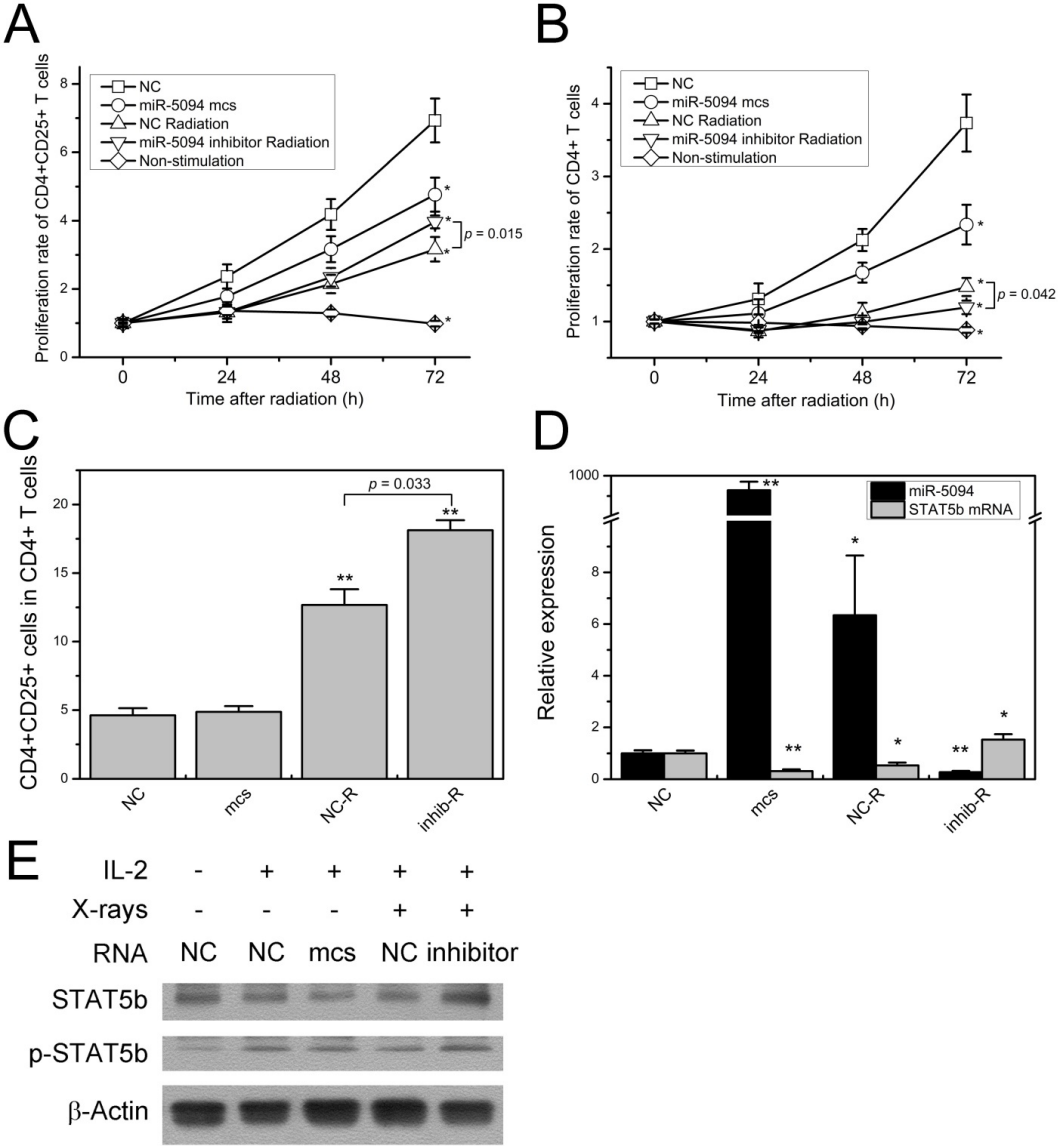
** miR-5094 regulates T cells proliferation by targeting STAT5b. (A)** Proliferation curves of CD4^+^CD25^+^ T cells from human Peripheral blood. **(B)** Proliferation curves of CD4^+^ T cells from human Peripheral blood. **(C)** Percentage of CD4^+^CD25^+^ T cells in CD4^+^ T cells at 72 h after radiation. **(D)** Expression of miR-5094 and STAT5b mRNA in CD4^+^ T cells at 24 h after radiation. U6 and GADPH mRNAs were used as controls. **(E)** Expression and activation of STAT5b in CD4^+^ T cells at 24 h after radiation. Cells were cultured with anti-CD3/CD28-coated beads (1:4) and subjected to IL-2 (20 U/mL) stimulation as indicated. NC: negative mock control; mcs: miR-5094 mimics; inhib: miR-5094 inhibitor; R: 2 Gy X-rays. *P < 0.05 and **P < 0.01 represent the comparison with NC.

As shown in Figure 5A, 2 Gy of X-rays significantly suppressed proliferation of CD4^+^CD25^+^ Treg cells. As compared with mock control transfection, proliferation of CD4^+^CD25^+^ Treg cells was remarkably decreased at 72 h after miR-5094 transfection. Moreover, transfection of miR-5094 inhibitor mitigated the radiation-induced decline of Treg cells proliferation. Similarly, miR-5094 transfection and X-ray irradiation suppressed proliferation of CD4^+^ T cells (Figure 5B). However, the transfection of miR-5094 inhibitor in CD4^+^ T cells did not rescue the compromised proliferation of CD4^+^ T cells after radiation. As expected, the proliferation of both CD4^+^CD25^+^ Treg cells and CD4^+^ T cells decreased significantly without stimulation from anti-CD3/CD28 and IL-2 (Figure 5A and 5B).

The detection of CD4^+^CD25^+^ Treg proportion in all CD4^+^ T cells showed that X-ray irradiation dramatically increased Treg proportions. More importantly, miR-5094 inhibitor further increased the percentage of CD4^+^CD25^+^ Treg than radiation treatment alone (Figure 5C).

The expression of STAT5b and miR-5094 in CD4^+^ T cells were next examined by qRT-PCR and Western blotting. The result showed that in CD4^+^ T cells, miR-5094 mimics or X-ray irradiation induced up-regulation of miR-5094 and down-regulation of STAT5b (Figure 5D). The miR-5094 inhibitor eliminated such an up-regulation or down-regulation (Figure 5D). Likewise, the Western blotting showed that the expression of STAT5b was suppressed by miR-5094 mimics or X-rays. Furthermore, the IR induced decrease in STAT5b was rescued by miR-5094 inhibitor. As expected, IL-2 stimulated the phosphorylation of STAT5b (Figure 5E).

## Discussion

Our previous study demonstrated that miR-5094 is a novel human miRNA and detected its up-regulation after 2-Gy X-ray irradiation by qRT-PCR using stem-loop reverse transcription primers 16. In this study, we confirmed responsive increase of miR-5094 in HeLa cells, Jurkat cells and human peripheral blood T cells after X-ray irradiation (Figure 2 and Figure 5). Additionally, we showed the expression levels of miR-5094 were elevated alongside with increasing X-ray dosage (Figure 2). These data verified miR-5094 is a radiation responsive miRNA and it is dramatically up-regulated by radiation treatment. Identifying target(s) of miR-5094, therefore, will advance current understanding of cellular radiation response.

Through TargetScan analysis, STAT5b was predicted as a putative target of miR-5094. Our reporter gene assay indeed showed that miR-5094 down-regulates reporter through the putative miR-5094 binding site in 3'-UTR of STAT5b. We further verified the suppression of STAT5b expression by miR-5094 mimics (Figure 1B and 1C). Moreover, our detection of miR-5094 and STAT5b expression at transcription and protein level after X-ray irradiation revealed an inversely correlation between STAT5b and miR-5094 (Figure 2A and 2B). These data suggested a regulatory link between miR-5094 and STAT5b.

It has been reported that STAT5b was also suppressed by other miRNAs, e.g., miR-134, miR-150-5p and miR-200a 30-32. Moreover, it was reported that the expression of miR-134 was specifically up-regulated in human B lymphoblastic cell line IM9 at 24 h after 1 Gy and 10 Gy γ-ray irradiation 34, and miR-200a showed a significant higher expression in patient saliva 12 months after radiotherapy 35. These miRNAs were putative regulators of STAT5b after IR. Yet, no investigations have linked the expression changes of these miRNA to the changes of STAT5b in radiation response. Whereas, the data presents here showed no significant increase of miR-134, miR-150-5p and miR-200a in HeLa cells at 12 h and 24 h after X-ray irradiation (Figure 2C). According to recent studies on profiles of radiation responsive miRNA, the association between miRNA regulation and radiation exposure was showed to be dependent on the cell types, dosages and times after irradiation 8, 36, 37. Likewise, Cha *et al*. reported 3.3-fold up-regulation of miR-134 and 2.6-fold down-regulation of miR-197 in IM9 cells at 24 h after 10 Gy γ-Ray irradiation 34, while they were not able to detect up-regulation of miR-134 but found 5.34-fold up-regulation of miR-197 in IM9 cells at 8 h after 10 Gy γ-Ray irradiation 38. The inconsistency between previous research and our current study of miR-134, miR-150-5p and miR-200a may be due to the differences in cell types and time points after radiation used in studies.

Through regulation the expression of down-stream genes, STAT5b plays crucial role in cell growth, apoptosis and cell cycle arrest 21-23, 31, 39, 40. Since these processes often take place during DDR, we hypothesized that miR-5094 may participates in cellular radiation response via STAT5b pathway. Our results showed that exogenous miR-5094 significantly suppressed cell proliferation of HeLa cells, Jurkat cells, CD4^+^CD25^+^ Treg cells and CD4^+^ T cells (Figure 4B, 4D, 5A and 5B). Meanwhile, the proliferation suppressing effect induced by X-ray irradiation were attenuated by miR-5094 inhibitor in HeLa cells, Jurkat cells, and CD4^+^CD25^+^ Treg cells (Figure 4B and 5A). Furthermore, we validated the down-regulation of STAT5b by miR-5094 resulted in subsequent down-regulation of Bcl-2 and Igf-1 expression in both transcriptional and translational levels in HeLa cells after X-ray irradiation, and the transfection of miR-5094 inhibitor rescued the miR-5094-mediated expression suppression of STAT5b, Bcl-2 and Igf-1 after irradiation (Figure 3). In the other hand, though the expression of Bcl-2, p21 and Cyclin D1 is changed in miR-5094 mimics transfection or miR-5094 inhibitor transfection groups (Figure 3B), we failed to detect remarkable increasing of G1 phase arrested cells nor apoptosis cells in miR-5094 mimics transfected groups (Figure 4C and 4D), and the miR-5094 inhibitor did not show a significant influence on cell cycle distributions after X-ray irradiation. The probable reason for this result might be the effect of miR-5094 on cell cycle or apoptosis is so weak and is covered up by the intensive influence from ionizing radiation. Taken together, our data revealed that miR-5094 play a suppressive role in cell proliferation by targeting STAT5b during cellular radiation response.

Notably, the proliferation suppression of CD4^+^ T cells was not recovered by miR-5094 inhibitor after radiation, while the proliferation of CD4^+^CD25^+^ Treg cells was significantly increased under same treatment (Figure 5A and 5B). To understand this phenomenon, we subsequently detected the distribution of CD4^+^CD25^+^ Treg cells in total CD4^+^ T cells. Results showed that radiation induced markedly increase of CD4^+^CD25^+^ Treg cells, while the miR-5094 inhibitor induced higher percentage of CD4^+^CD25^+^ Treg cells after radiation (Figure 5C). These data coincident with previous studies that ionizing radiation impaired totally number of CD4^+^ T cells and CD4^+^CD25^+^ Treg cells 41, 42, while the ratio of Treg to CD4^+^ T cells is significantly enhanced as CD4^+^CD25^+^ Treg cells are more resistant to ionizing radiation than other T cells 41-43. Additionally, though the immune suppression function of CD4^+^CD25^+^ Treg cells was impaired by radiation, the extent of the reduction was limited especially at lower dose according to different studies 41, 42, 44, 45. Thus, the enhanced radiation sensitivity of total CD4^+^ T cells in miR-5094 inhibition group may contributes to a higher percentage of CD4^+^CD25^+^ Treg cells after radiation (Figure 5C), which in turn suppressed the proliferation of CD4^+^CD25^-^ T cells. As the immunosuppressing effects of Treg cells always considered as side effect of radiation therapy 46, our data indicates miR-5094 may participates in the maintenance of immunosuppressing effects of Treg after IR. Altogether, our study uncovered the proliferation suppression function of radiation induced miR-5094 via STAT5b in CD4^+^CD25^+^ Treg cells* in vitro*. Considering the difference between *in vivo* and *in vitro* system, further functional analysis will be necessary to elucidate the role of miR-5094 in Treg cells and immune system under ionizing radiation condition and we will work on it.

In summary, our study uncovered a role of radiation responsive miR-5094 in negative regulation of STAT5b protein level via miR-5094 binding site in STAT5b 3'-UTR. Our study also revealed that in cellular radiation response, negative regulation of STAT5b miR-5094 leads to subsequent inhibition of cell proliferation through the suppressing the transcription of STAT5b down-stream genes Bcl-2 and Igf-1. Our study provides a new insight into the role of miRNAs in STAT5b regulatory network in response to IR.
